# Structural and Signaling Events Driving *Aspergillus fumigatus*-Induced Human Eosinophil Extracellular Trap Release

**DOI:** 10.3389/fmicb.2021.633696

**Published:** 2021-02-18

**Authors:** Marina Valente Barroso, Isabella Gropillo, Marcella A. A. Detoni, Glaucia A. Thompson-Souza, Valdirene S. Muniz, Claudia Regina Isaías Vasconcelos, Rodrigo T. Figueiredo, Rossana C. N. Melo, Josiane S. Neves

**Affiliations:** ^1^Institute of Microbiology Paulo de Góes, Federal University of Rio de Janeiro, Rio de Janeiro, Brazil; ^2^Institute of Biomedical Sciences, Federal University of Rio de Janeiro, Rio de Janeiro, Brazil; ^3^Institute of Biomedical Sciences/Campus of Duque de Caxias, Federal University of Rio de Janeiro, Rio de Janeiro, Brazil; ^4^Laboratory of Cellular Biology, Department of Biology, Institute of Biological Sciences, Federal University of Juiz de Fora, Juiz de Fora, Brazil

**Keywords:** eosinophils, *A. fumigatus*, extracellular DNA traps, allergic bronchopulmonary aspergillosis, ABPA

## Abstract

Eosinophils are granulocytes classically involved in allergic diseases and in the host immune responses to helminths, fungi, bacteria and viruses. The release of extracellular DNA traps by leukocytes is an important mechanism of the innate immune response to pathogens in various infectious conditions, including fungal infections. *Aspergillus fumigatus* is an opportunistic fungus responsible for allergic bronchopulmonary aspergillosis (ABPA), a pulmonary disease marked by prominent eosinophilic inflammation. Previously, we demonstrated that isolated human eosinophils release extracellular DNA traps (eosinophil extracellular traps; EETs) when stimulated by *A. fumigatus in vitro*. This release occurs through a lytic non-oxidative mechanism that involves CD11b and Syk tyrosine kinase. In this work, we unraveled different intracellular mechanisms that drive the release of extracellular DNA traps by *A. fumigatus*-stimulated eosinophils. Ultrastructurally, we originally observed that *A. fumigatus*-stimulated eosinophils present typical signs of extracellular DNA trap cell death (ETosis) with the nuclei losing both their shape (delobulation) and the euchromatin/heterochromatin distinction, followed by rupture of the nuclear envelope and EETs release. We also found that by targeting class I PI3K, and more specifically PI3Kδ, the release of extracellular DNA traps induced by *A. fumigatus* is inhibited. We also demonstrated that *A. fumigatus*-induced EETs release depends on the Src family, Akt, calcium and p38 MAPK signaling pathways in a process in which fungal viability is dispensable. Interestingly, we showed that *A. fumigatus*-induced EETs release occurs in a mechanism independent of PAD4 histone citrullination. These findings may contribute to a better understanding of the mechanisms that underlie EETs release in response to *A. fumigatus*, which may lead to better knowledge of ABPA pathophysiology and treatment.

## Introduction

Eosinophils are bone marrow-derived granulocytes that are typically abundant in inflammatory infiltrates in the defense against helminthic parasites and in allergic diseases. However, various immunoregulatory actions and functions have been also described for eosinophils, such as lymphocyte recruitment, tissue repair, organ development, antigen presentation, antimicrobial and antifungal activities, among other functions (Strandmark et al., 2016). Two of the main eosinophil activation mechanisms are degranulation and, more rarely, phagocytosis (Shamri et al., 2011). Recently, another cell effector mechanism has been described for eosinophils—the release of extracellular DNA traps (Yousefi et al., 2008; Ueki et al., 2013). The release of extracellular DNA traps (ETs) by leukocytes has been considered an important mechanism of the immune response in different inflammatory conditions. Although most ET knowledge is based on neutrophil studies (Brinkmann et al., 2004; Fuchs et al., 2007; Parker et al., 2012), it is well known that other leukocytes are also able to release ETs, including eosinophils (Yousefi et al., 2008), mast cells (von Kockritz-Blickwede et al., 2008), monocytes/macrophages (Chow et al., 2010), dendritic cells (Ramirez-Ortiz et al., 2011; Loures et al., 2015) and basophils (Schorn et al., 2012; Morshed et al., 2014). Various stimuli are capable of inducing the release of ETs, which essentially are structures of disrupted chromatin filaments coated with granular and cytosolic proteins, histones, and proteases (Brinkmann et al., 2004; Fuchs et al., 2007; Guimaraes-Costa et al., 2009; Parker et al., 2012). ET release involves non-lytic (Yousefi et al., 2008, 2009; Yipp et al., 2012) or lytic processes (named ETosis) that in general require cell chromatin decondensation (Brinkmann et al., 2004; Fuchs et al., 2007; Guimaraes-Costa et al., 2009; Parker et al., 2012; Ueki et al., 2013). Some studies indicate that this process is due to histone hypercitrullination by the enzyme PAD4 (peptidylarginine deiminase 4), in which arginine residues in histones are converted to citrulline, allowing DNA fibers to unfold (Wang et al., 2009; Li et al., 2010; Lewis et al., 2015; Van Avondt and Hartl, 2018). However, controversy remains about the relative importance of PAD4 for ETs release (Neeli and Radic, 2013; Kenny et al., 2017; Claushuis et al., 2018; Guiducci et al., 2018; Silva et al., 2019; Thompson-Souza et al., 2020). Interestingly, some studies have indicated that mitochondria are the source of the DNA that composes ETs (Yousefi et al., 2008, 2009). ETs help leukocytes immobilize bacteria, fungi and viruses, creating a microenvironment that favors a more efficient elimination of pathogens (Brinkmann et al., 2004; Fuchs et al., 2007; Papayannopoulos et al., 2010; Parker et al., 2012). However, recent evidence has emerged suggesting that ETs also have a role in non-infectious sterile inflammation (Jorch and Kubes, 2017). In eosinophils, the process by which they produce and release eosinophil extracellular DNA traps (EETs) can result in cell death (named EETosis in eosinophils) (Ueki et al., 2013); or in a non-lytic process by which eosinophils rapidly produce EETs but do not lose their viability (Yousefi et al., 2008, 2009).

Exposure and sensitization to fungal allergens is an important factor in patients with respiratory allergies; in this context, fungi play an important role in the development, severity and persistence of allergic lung diseases (Knutsen et al., 2012; Denning et al., 2014). Allergic bronchopulmonary mycoses (ABPMs) are characterized by fungal colonization and are known to worsen lung function, which is commonly associated with the development of severe asthma (Denning et al., 2013, 2014; Chowdhary et al., 2014). *Aspergillus fumigatus* is the most common cause of ABPMs; to a lesser extent, *C. albicans* and *Alternaria* species are also related to the development of these diseases (Shah and Panjabi, 2002; Chowdhary et al., 2014). ABPA, a form of non-invasive eosinophilic pulmonary aspergillosis, is a multifaceted pulmonary disorder caused by immunological reactions in response to repeated antigen exposure and/or colonization by *A. fumigatus* (Agarwal et al., 2013; Ueki et al., 2018). Susceptibility is related to the pathophysiology of comorbidities such as asthma, sinusitis, cystic fibrosis and alveolitis (Kousha et al., 2011; Denning et al., 2014). We recently described the presence of EETs in sputum samples from ABPA patients and found that human eosinophils are capable of releasing EETs *in vitro* in response to *A. fumigatus* conidia in a lytic non-oxidative process that involves CD11b and the Syk signaling pathway (Muniz et al., 2018). However, the ultrastructural features and signaling events that characterize and drive *A. fumigatus*-induced EETosis in human eosinophils are not completely understood. Thus, the purpose of this study was to unravel the intracellular mechanisms that direct the process of extracellular DNA trap release by *A. fumigatus*-stimulated eosinophils.

## Methodology

### Study Approval

All protocols and experimental procedures that involved human blood-isolated eosinophils were approved by the Committee on Human Research at Clementino Fraga Filho Hospital (Federal University of Rio de Janeiro). Written informed consent was obtained under institutional review board approved protocols (license number CAAE 31968020.9.0000.5257).

### Fungal Culture and Conidial Preparation

*A. fumigatus* conidia ATCC 46645 (strain NCPF 2109) cryopreserved in liquid nitrogen and maintained in medium that contained 0.9% saline, 0.01% Tween, and 30% glycerol was thawed and spread onto solid potato dextrose agar medium (Neogen, MI, United States). The culture was incubated for 5–7 days at 37°C. Plates that contained mycelium of *A. fumigatus* were scraped with sterile PBS containing 0.05% Tween-20 (Bio-Rad, CA, United States), and the conidia were collected via filtration through a sterile nylon mesh with a porosity of 40 μm (BD Biosciences, NJ, United States). The conidia were pelleted by centrifugation (3150 *g*, 25°C, 15 min), resuspended in RPMI 1640 (phenol red-free) (Sigma, MO, United States), and counted using a Neubauer chamber and an optical microscope (Leica Microsystems, Wetzlar, Germany) at 40× magnification. The cell concentration was adjusted for the subsequent stimulation experiments. For a specific set of experiments, *A. fumigatus* conidia were fixed in 4% paraformaldehyde (PFO) for 30 min at room temperature, extensively washed with PBS (2 ml, three times), centrifuged (3160 *g*, 15 min) and resuspended in RPMI.

### Eosinophil Purification

Eosinophils were isolated from the blood of healthy donors using negative selection as previously described (Muniz et al., 2018). The viability and purity of the freshly isolated eosinophils were more than 99%, as analyzed by trypan blue exclusion and panoptic kit staining, respectively.

### Fluorimetric Assay for EET Quantification

Purified human eosinophils (2 × 10^5^/200 μL) were resuspended in RPMI 1640 (phenol red-free) supplemented with 0.1% heat-inactivated fetal calf serum (Life Technologies, CA, United States), 1% L-glutamine (Life Technologies) and antibiotics (penicillin and streptomycin). The eosinophils were stimulated in 96-well tissue culture plates with *A. fumigatus* conidia at a cell:fungus ratio of 1:10 for 6 h at 37°C, conditions that were previously determined in previous studies (Muniz et al., 2018). Ten minutes before the end of the incubation time, Sytox Green (5 μM, Life Technologies), an extracellular DNA probe impermeable to viable cells, was added to the wells. The samples were analyzed in a FlexStation plate reader (Molecular Devices, CA, United States) with a wavelength combination of excitation at 485 nm and emission at 538 nm. The values are expressed in relative fluorescence units. To evaluate the participation of different signaling pathways in *A. fumigatus*–induced release of EETs, the eosinophils were pretreated for 30 min before fungal stimulation with the following inhibitors: PP2 (10 μM, Caymann, MI, United States), a pharmacological inhibitor for Src kinases; wortmannin (100 ηM, Sigma), a PI3K pan-inhibitor; Akt inhibitor VIII (2.6 μM, Cayman), an Akt inhibitor; SB202190 (10 μM, Calbiochem, CA, United States), a p38 MAPK inhibitor; AS605240 (Cayman), at 10 μM, a selective inhibitor of the class I PI3K family; IC-87114 (Cayman), at 1 μM, a selective PI3K δ inhibitor; GSK484 (10 μM, GlaxoSmithKline, Brentford, United Kingdom), a PAD4 inhibitor; and BAPTA-AM (10 μM, Sigma), a calcium chelating agent. In all conditions, the inhibitor vehicle, dimethyl sulfoxide (DMSO), was tested at the corresponding dilution. 0

### Confocal Microscopy

Purified human eosinophils (2 × 10^5^/1000 μL) were placed in a 24-well plate that contained coverslips pretreated with poly-L-lysine (0.001%) (Sigma). Before fungal stimulation, the eosinophils were pretreated for 30 min with the pharmacological inhibitors described above. The cells were subsequently stimulated with *A. fumigatus* conidia at a cell: fungus ratio of 1:10 and were maintained at 37°C with 5% CO_2_ for 6 h. At the end of the incubation time, the culture medium was removed, and the adhered cells were fixed with 4% PFO for 15 min at room temperature (RT). The cells were washed three times with sterile PBS. Sytox Green (5 μM) or Hoechst (1:1000, Life Technologies) was subsequently added and incubated for 10 min for DNA labeling. For histone labeling, the samples were fixed with 4% PFO and permeabilized with PBS buffer containing 1% Triton X-100 and 2% NP40. They were then labeled with a mouse anti-human citrullinated histone H3 antibody (0.8 μg/mL, Abcam, Cambridge, United Kingdom) diluted in PBS containing 0.05% Tween-20, 2% BSA, and 5% human serum. Following an overnight incubation at 4°C, the cells were washed and incubated for 1 h with a rabbit anti-mouse IgG fluorescein isothiocyanate–conjugated antibody (1:1000, Jackson ImmunoResearch, ME, United States). The wells were then washed 3 times, followed by the addition of the mounting medium Aqua-Poly/Mount. Images were acquired using a fluorescence confocal microscope (Leica TCS SP5, Leica Microsystems, Wetzlar, Germany) and were analyzed with the ImageJ program (Fiji).

### Immunoblotting

Purified human eosinophils (1 × 10^6^/200 μL) were placed in a 24-well plate and pretreated for 30 min with the PAD4 inhibitor GSK484 (10 μM, GlaxoSmithKline) or its vehicle and then stimulated with *A. fumigatus* conidia (cell: fungus ratio of 1:10, 37°C, 6 h). After incubation, the cells were lysed in sample buffer (Tris HCl 62 mM, 10% glycerol, 5% β-mercaptoethanol, 2% SDS). The samples were boiled (100°C for 5 min), centrifuged to remove insoluble debris, run on a polyacrylamide gel (12%), and then transferred to a nitrocellulose membrane using a Trans-Blot Semi-Dry Transfer Cell (Bio-Rad). The membranes were blocked with TBS-T (Tris-buffered saline + 0.05% Tween) containing 5% BSA and then incubated overnight with a primary rabbit anti-human citrullinated histone H3 polyclonal antibody (0.8 μg/mL, Abcam) or a rabbit anti-human histone H3 monoclonal antibody (clone D1H2, Cell Signaling, Massachusetts, United States). The membranes were subsequently incubated for 1 h with a secondary goat anti-rabbit IgG peroxidase-conjugated antibody (Sigma) and were revealed by chemiluminescence (ECL, Thermo Fisher Scientific, MA, United States).

### Transmission Electron Microscopy

Blood eosinophils were added to chamber slides (2.5 ×10^5^/chamber), stimulated or not with A. fumigatus conidia (cell: fungus ratio of 1:10) for 6 h, and immediately fixed in a mixture of freshly prepared aldehydes (2.5% paraformaldehyde, 2.5% glutaraldehyde) in 1 M sodium cacodylate buffer, pH 7.4, for 30 min at room temperature. To allow optimal cell morphology and observation of extracellular trap formation in situ, all electron microscopy procedures were performed at RT directly on the slide surface as previously described (Ueki et al., 2013). The cells were post-fixed in 1% osmium tetroxide and processed for transmission electron microscopy (TEM) according to previous studies (Melo et al., 2009). Resin embedding was performed by inverting resin-filled plastic capsules over the slide-attached cells. After polymerization at 60°C for 16 h, thin sections were cut using a diamond knife on an ultramicrotome (Leica, Bannockburn, IL, United States). Sections were mounted on uncoated 200-mesh copper grids (Ted Pella, California, United States) before staining with lead citrate and then viewed with a transmission electron microscope (Tecnai G2 Spirit, FEI/Thermo Fisher Scientific) at 80 kV. A total of 77 randomly acquired electron micrographs were analyzed at different magnifications.

### Scanning Electron Microscopy (SEM)

Purified human eosinophils (2 × 10^5^/200 μL) were placed in a 24-well plate that contained coverslips pretreated with poly-L-lysine (0.001%) (Sigma). *A. f*umigatus conidia were added at a cell: fungus ratio of 1:10, and the samples were maintained at 37°C with 5% CO_2_. After 6 h of incubation, the culture medium was removed, and cells that adhered to the coverslip were fixed with 2.5% glutaraldehyde, 4% formaldehyde (0.1 M) in sodium cacodylate buffer for 2 h at RT. After three washes in sodium cacodylate buffer (0.1 mol/L), the samples were post-fixed with 1% osmium tetroxide and 0.8% potassium ferrocyanide in sodium cacodylate buffer for 30 min. The samples were again washed three times in sodium cacodylate buffer, followed by dehydration in graded ethanol: 30, 50, 70, 90, and 100% for 15 min each. The critical point technique with CO_2_ was subsequently performed, followed by mounting on a metallic support with carbon tape. The samples were then covered with a thin layer of 20 nm gold (metallization), followed by examination under a conventional QUANTA 250 FEI scanning electron microscope (ThermoFisher Scientific).

### Statistical Analysis

The results were analyzed with GraphPad Prism 8 using ANOVA with repeated measures and the Newman-Keuls post-test, with statistically significant differences defined as *p* < 0.05.

## Results

### *A. fumigatus*-Induced EETosis Is Characterized by Marked Nuclear Alterations

**FIGURE 1:**
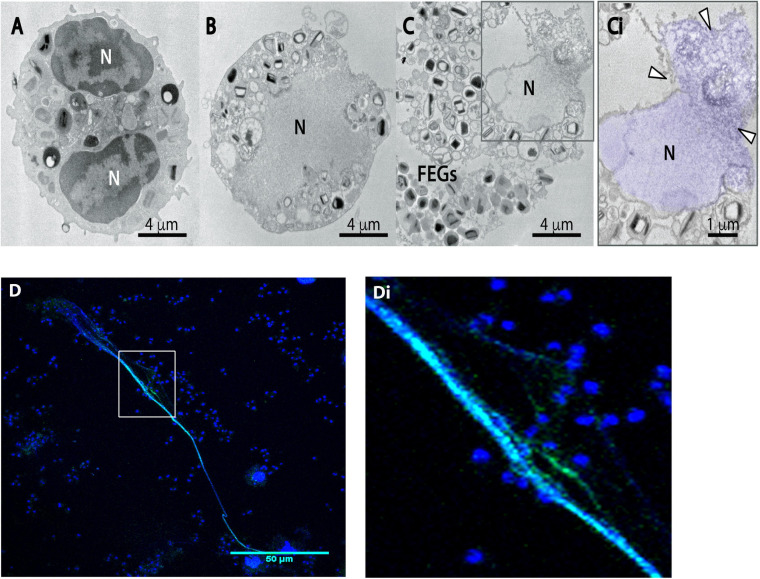
Human eosinophils exposed to *A. fumigatus* show typical EETosis-associated nuclear changes and release chromatin-citrullinated histone EETs. Human eosinophils were stimulated with *A. fumigatus* conidia (cell:fungus ratio of 1:10) for 6 h **(A)** A representative unstimulated eosinophil with a typical bilobulated nucleus (N) and well-defined euchromatin and heterochromatin. **(B,C,Ci)** After interaction with the fungal conidia, eosinophil nuclei undergo delobulation, disintegration of the nuclear envelope, chromatin decondensation/expansion (colored in purple) and release of extracellular traps [arrowheads in panel **(Ci)**]. Most free extracellular granules (FEGs) maintain their morphology. **(D)** EETs and citrullinated histone colocalization are shown by confocal fluorescence microscopy after staining for DNA (Hoechst, blue) and anti-citrullinated H3 histone antibodies (green). Panel **(Di)** is the boxed area at a higher magnification. Representative images of 3 independent experiments (*n* = 3).

To investigate the intracellular structural events associated with the formation of EETs by human eosinophils in response to *A. fumigatus*, cells incubated with this pathogen in chamber slides were fixed and processed for TEM directly on the slide surface, without any additional procedures that could interfere with the cell morphology. This ultrastructural analysis revealed for the first time that eosinophils undergo marked EETosis-associated nuclear alterations upon interaction with *A. fumigatus*. The typically bilobed nuclei with well-defined euchromatin/heterochromatin areas as seen in control cells (Figure 1A) lost their shape in parallel with chromatin decondensation/expansion, and the distinction between euchromatin/heterochromatin disappeared (Figure 1B). Rupture of the nuclear envelope and plasma membrane (Figure 1C) allowed the release of chromatin-formed EETs (arrowheads in Figure 1Ci) from cytolytic eosinophils and free extracellular granules (FEGs) (Figure 1C). Accordingly, DNA-citrullinated histone EETs released in response to *A. fumigatus* were consistently immunolabeled (Figures 1D,Di), as previously demonstrated (Muniz et al., 2018).

**FIGURE 2:**
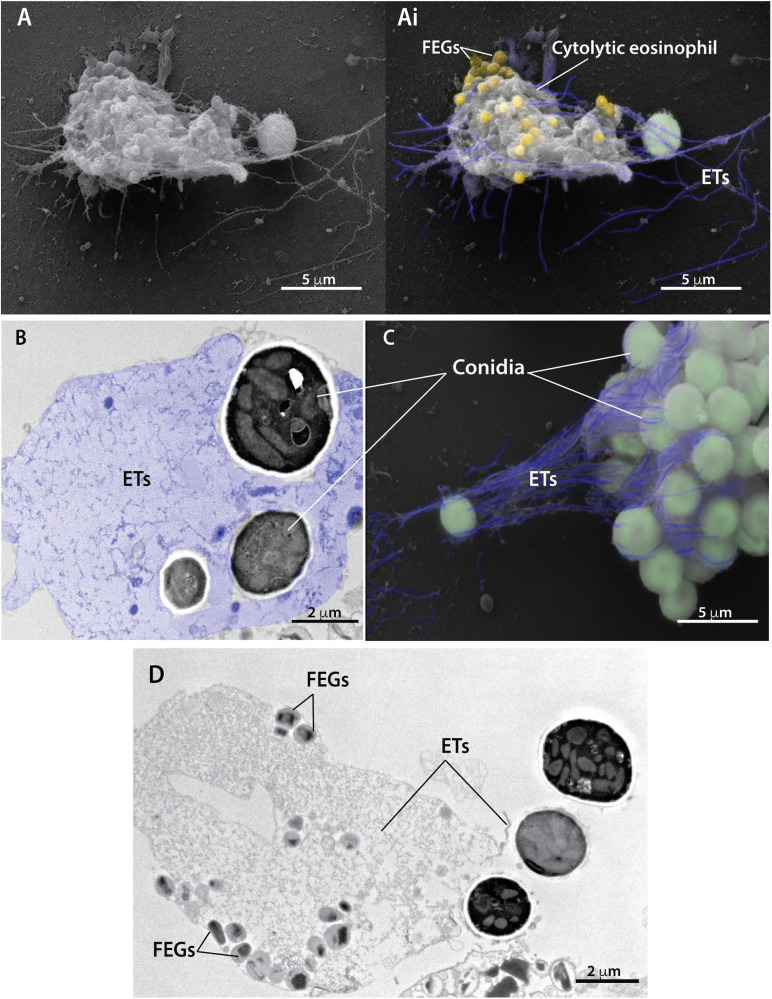
Extracellular traps released from cytolytic eosinophils entrap conidia from *A. fumigatus*. Human eosinophils were stimulated with *A. fumigatus* conidia (cell: fungus ratio of 1:10) for 6 h and processed for conventional TEM and SEM. **(A,Ai,C)** SEM and **(B,D)** TEM revealed eosinophil extracellular traps (ETs) composed of fibers of DNA (highlighted in purple) emerging from cytolytic human eosinophils and entrapping conidia (colored in green). Note in a thin section (**B**) that the fungal cell wall is completely surrounded by eosinophil ETs. Eosinophil ETs are decorated with free extracellular granules (FEGs) seen in both 3D [Panels **(A,Ai)** in yellow] and two dimensions **(D)**. The typical eosinophil granule ultrastructure with an electron-dense core and less dense matrix is observed (**D**).

Analyses at high resolution by both SEM (Figures 2A,Ai,C) and TEM (Figures 2B,D) revealed the ultrastructure of EETs being released from cytolytic eosinophils. In three dimensions (3D), EETs appeared as typical elongated DNA fibers (Figures 2A,Ai), while *in situ*, TEM images of adhered cells showed the two-dimensional appearance of the extruded EETs, which covered large areas outside the cells (Figures 2B colored in purple, 2D). *A. fumigatus* conidia entrapped by EETs were observed in 3D (Figures 2A,Ai,C colored in green) and in thin sections by TEM, which revealed that the fungus cell wall was completely involved in the DNA ETs (Figures 2B,D). Free extracellular granules (FEGs) with preserved limiting membranes and typical morphology represented by an electron-dense core surrounded by an electron-lucent matrix were frequently observed in association with cytolytic eosinophils, thus demonstrating that the EETosis triggered by *A. fumigatus* leads to the release of nearly intact granules (Figures 2Ai in yellow, 2D). Quantitative TEM of 54 randomly acquired cell sections demonstrated that most eosinophils (87.2%) in interaction with *A. fumigatus* were cytolytic, with morphological changes typical of EETosis.

### Release of EETs in Response to *A. fumigatus* Involves Src Kinase Family Activation

**FIGURE 3:**
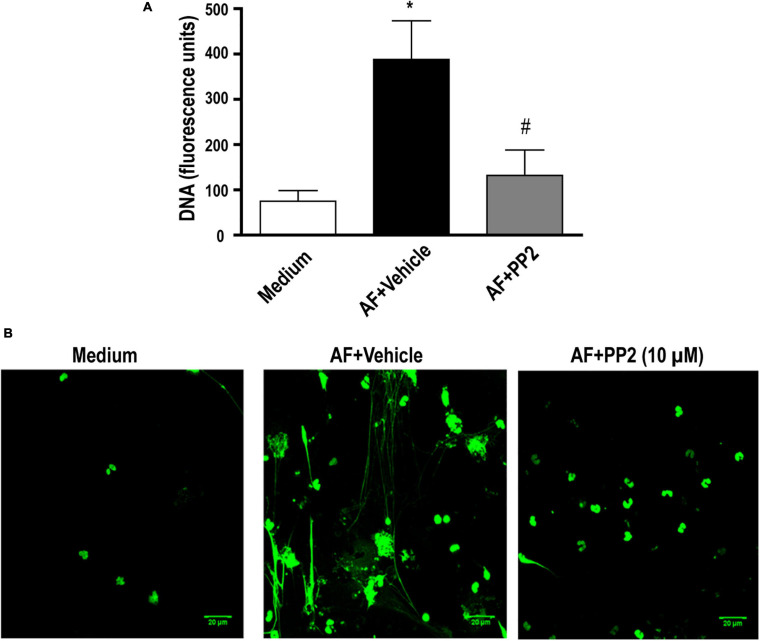
*A. fumigatus*-induced EETs release depends on the Src kinase family. Human eosinophils were pretreated with a Src kinase family inhibitor (PP2) (10 μM), or its vehicle (DMSO) for 30 min and were subsequently stimulated with *A. fumigatus* conidia (cell: fungus ratio of 1:10) for 6 h. **(A)** EETs were quantified in the samples using a fluorescence microplate reader after Sytox Green addition (5 μM). The graph represents the mean ± SEM of 4 independent experiments from different donors. *P < 0.05 compared to the non-stimulated sample (medium); #*P* < 0.05 compared to AF + vehicle; one-way ANOVA followed by the Newman-Keuls test. **(B)** Confocal fluorescence microscopy of human eosinophils pretreated with PP2 (10 μM) or its vehicle (DMSO) for 30 min and subsequently stimulated with *A. fumigatus* conidia (cell: fungus ratio of 1:10) for 6 h after staining for DNA (Sytox Green, green). Representative images of 4 independent experiments (*n* = 4). AF = *Aspergillus fumigatus.*

We previously showed that *A. fumigatus*-induced EETs release is dependent on integrin CD11b and Syk kinases (Muniz et al., 2018). Syk and Src kinases have been shown to mediate cell signaling via different classes of receptors involved in fungal recognition, including integrins (Mocsai et al., 2002, 2006). Thus, the role of Src kinases in *A. fumigatus-*induced EETs release was investigated. In the presence of PP2 (10 μM), which is known to inhibit a broad spectrum of Src kinases, the release of extracellular DNA traps by eosinophils was abolished (Figure 3A). Confocal fluorescence microscopy confirmed these results (Figure 3B).

### Release of EETs in Response to *A. fumigatus* Requires the PI3 Kinase, Akt and p38-MAPK Signaling Pathways

**FIGURE 4:**
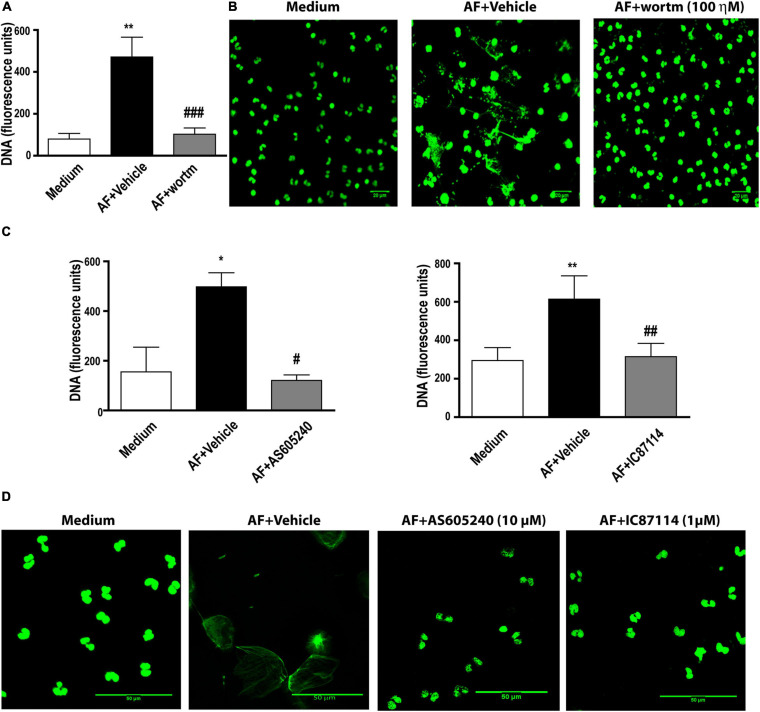
*A. fumigatus*-induced EETs release requires PI3 kinase activation. Human eosinophils were pretreated with **(A,B)** the pan-PI3K inhibitor wortmannin (wortm – 100 ηM), **(C,D)** a class I PI3K inhibitor (AS605240 – 10 μM) and a PI3Kδ inhibitor (IC87114 – 1 μM), or their vehicle (DMSO), for 30 min and were subsequently stimulated with *A. fumigatus* conidia (cell: fungus ratio of 1:10) for 6 h. **(A,C)** EETs were quantified in the samples using a fluorescence microplate reader after Sytox Green addition (5 μM). The graphs represents the mean ± SEM of 3 (wortm), 3 (AS605240) and 7 (IC87114) independent experiments from different donors. *P < 0.05 and **P < 0.01 compared to the non-stimulated sample (medium); # *P* < 0.05, ##*P* < 0.01, ### *P* < 0.001 compared to AF + vehicle condition; one-way ANOVA followed by the Newman-Keuls test. **(B,D)** Confocal fluorescence microscopy of human eosinophils pretreated with **(B)** 100 ηM wortmannin, **(D)** 10 μM AS605240 and 1 μM IC87114 or their respective vehicle dilutions (DMSO) for 30 min and subsequently stimulated with *A. fumigatus* conidia (cell: fungus ratio of 1:10) for 6 h after staining for DNA (Sytox Green, green). Representative images of 3 (wortm), 3 (AS605240) and 7 (IC87114) independent experiments (*n* = 3, 3 and 7). AF = *Aspergillus fumigatus.*

PI3K has been described as involved in the process of extracellular DNA trap release in neutrophils and eosinophils in response to different stimuli (Behnen et al., 2014; DeSouza-Vieira et al., 2016; Germic et al., 2017; Silva et al., 2019). Using fluorimetry (Figure 4A) and confocal fluorescence microscopy (Figure 4B), we observed that in the presence of 100 ηM wortmannin, a pan-PI3K inhibitor, EETs release in response to *A. fumigatus* was abolished. Class IA PI3K has been described as important for neutrophil extracellular DNA trap (NETs) (DeSouza-Vieira et al., 2016; Silva et al., 2019) and has been implicated as critical in different eosinophil responses (Kang et al., 2012; Saito et al., 2014). More specifically, class I PI3K δ has been described as critical for neutrophil extracellular trap release in response to *A. fumigatus* conidia (Silva et al., 2019) and *Leishmania* (DeSouza-Vieira et al., 2016), as well as for eosinophil trafficking, migration and morphology (Kang et al., 2012). Thus, the role of the class I PI3K family and the class I PI3K δ isoform in EETs release in response to *A. fumigatus* was assessed. As wortmannin does not distinguish among PI3K classes, we subsequently used the compound AS605240, which at 10 μM is an inhibitor of class I PI3K. We verified that 10 μM AS605240 completely inhibited EETs release (Figure 4C, left panel). Confocal fluorescence microscopy studies in which the samples were stained for DNA (Sytox Green, green) confirmed these findings (Figure 4D). In the presence of a selective PI3Kδ inhibitor (1 μM IC87114), we observed that the release of EETs in *A. fumigatus*-stimulated human eosinophils was inhibited (Figures 4C, right panel and 4D), suggesting that the process is dependent on the PI3K p110δ subunit.

**FIGURE 5:**
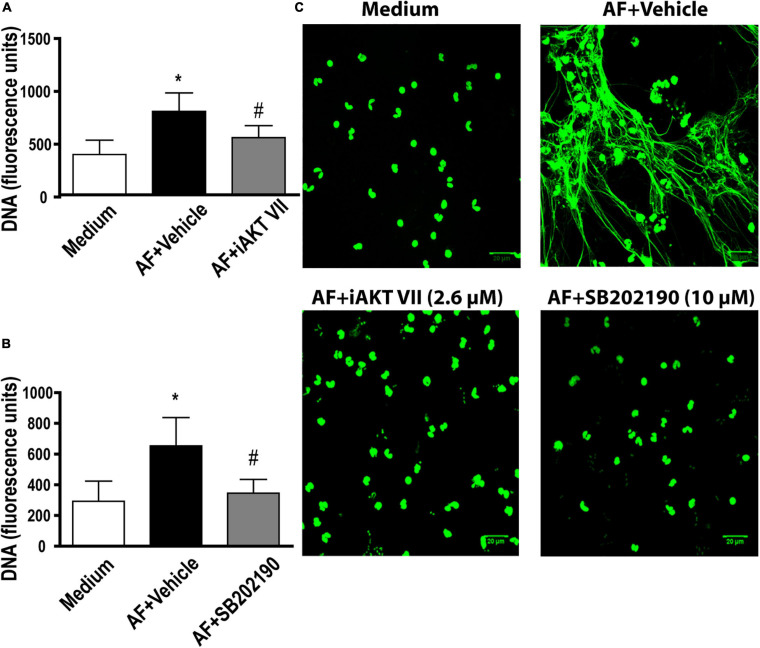
*A. fumigatus*-induced EETs release involves Akt and p38-MAPK signaling pathways. Human eosinophils were pretreated with **(A,C)** an Akt inhibitor (iAkt VIII – 2.6 μM) and **(B,C)** a p38-MAPK inhibitor (SB202190 – 10 μM) or their vehicles (DMSO) for 30 min and were subsequently stimulated with *A. fumigatus* conidia (cell: fungus ratio of 1:10) for 6 h. **(A,B)** EETs were quantified in the samples using a fluorescence microplate reader after Sytox Green addition (5 μM). The graphs represent the mean ± SEM of 3 **(A)** and 4 **(B)** independent experiments from different donors. *P < 0.05 compared to the non-stimulated sample (medium); # *P* < 0.05 compared to AF + vehicle; one-way ANOVA followed by the Newman-Keuls test. Samples were analyzed by **(C)** confocal fluorescence microscopy after staining for DNA (Sytox Green, green). Representative images of 3 (iAkt) and 4 (SB202190) independent experiments (*n* = 3 and *n* = 4). AF = *Aspergillus fumigatus.*

Akt kinase is commonly observed as a downstream activation molecule in the class I PI3K pathway (Hawkins et al., 2010). In addition, the participation of Akt has been demonstrated in the context of NETs release following immunocomplex recognition and signaling via Mac-1 (Behnen et al., 2014). Thus, we aimed to investigate the role of Akt in EETs release in response to *A. fumigatus*. As observed by fluorimetry (Figure 5A) and confocal fluorescence microscopy (Figure 5C), when we blocked the Akt signaling pathway via its inhibitor (iAkt VIII, 2.6 μM), the eosinophils did not release EETs.

p38-MAPK is known to be involved in the release of NETs in response to immune complexes (Behnen et al., 2014), calcium ionophores and PMA (Douda et al., 2015). Therefore, we investigated whether p38-MAPK was involved in *A. fumigatus*-induced EETs release. We found that compound SB202190 (10 μM) inhibited the process of EETs release (Figure 5B). Confocal fluorescence microscopy confirmed the fluorimetric findings (Figure 5C).

### Release of EETs in Response to *A. fumigatus* Requires Calcium

**FIGURE 6:**
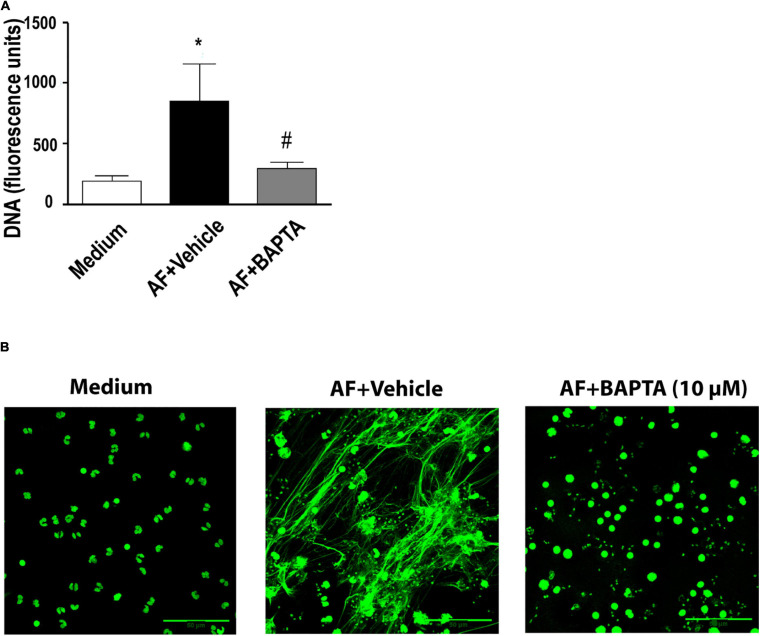
*A. fumigatus*-induced release of EETs depends on calcium. Human eosinophils were pretreated with the calcium chelator BAPTA-AM (10 μM) or its vehicle (DMSO) for 30 min and were subsequently stimulated with *A. fumigatus* conidia (cell: fungus ratio of 1:10) for 6 h. **(A)** EETs were quantified in the samples using a fluorescence microplate reader after Sytox Green addition (5 μM). The graph represents the mean ± SEM of 3 independent experiments from different donors. *P < 0.05 compared to the non-stimulated sample (medium); #*P* < 0.05 compared to AF + vehicle; one-way ANOVA followed by the Newman-Keuls test. **(B)** Confocal fluorescence microscopy of human eosinophils pretreated with BAPTA-AM (10 μM) or its vehicle (DMSO) for 30 min and subsequently stimulated with *A. fumigatus* conidia (cell: fungus ratio of 1:10) for 6 h after staining for DNA (Sytox Green, green). Representative images of 4 independent experiments (*n* = 4). AF = *Aspergillus fumigatus.*

Calcium increase in leukocytes is intimately associated with the pro-inflammatory functions of these cells (Dixit and Simon, 2012). Therefore, we tested the impact of the calcium chelator BAPTA-AM on the process of *A. fumigatus*-induced EETs release. We observed a complete inhibition of EETs release (Figure 6A). Accordingly, fluorescence microscopy confirmed the prevention of fungal-induced EETs extrusion (Figure 6B).

### PAD4-Mediated Histone Citrullination Is Dispensable for *A. fumigatus*-Induced EETs Release

**FIGURE 7:**
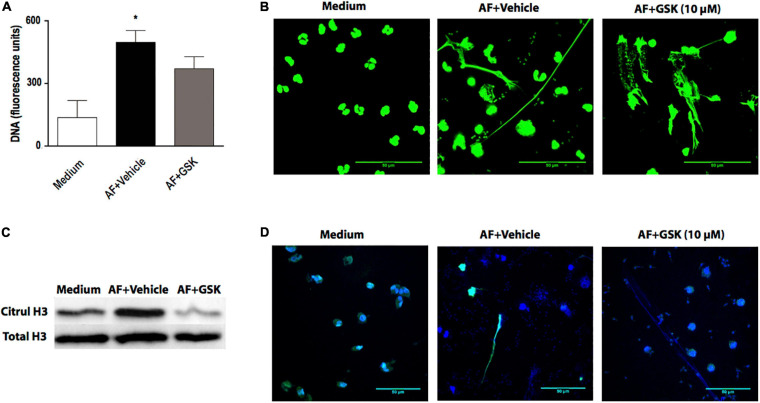
Histone citrullination mediated by PAD4 is dispensable for *A. fumigatus*-induced EETs release. Human eosinophils were pretreated with the PAD4 selective inhibitor GSK484 (10 μM) or its vehicle (DMSO) for 30 min and were subsequently stimulated with *A. fumigatus* conidia (cell: fungus ratio of 1:10) for 6 h. **(A)** EETs were quantified in the samples using a fluorescence microplate reader after Sytox Green addition (5 μM). The graph represents the mean ± SEM of 3 independent experiments from different donors. *P < 0.05 compared to the non-stimulated sample (medium); one-way ANOVA followed by the Newman-Keuls test. **(B)** Samples were analyzed by confocal fluorescence microscopy after staining for DNA (Sytox Green, green). Representative images of 3 independent experiments (*n* = 3). Histone citrullination was evaluated by **(C)** immunoblotting and **(D)** confocal fluorescence microscopy after staining for DNA (Hoechst, blue) and anti-citrullinated H3 histone antibodies (green). Representative immunoblotting images of 3 independent experiments (*n* = 3). AF = *Aspergillus fumigatus.*

In this study (Figure 1D) and in a previous study (Muniz et al., 2018), we showed that EETs released in response to *A. fumigatus* were associated with citrullinated histone H3. Histone citrullination is considered to play an essential role in the nuclear-derived EET formation mediated by the action of PAD4 (Li et al., 2010; Rohrbach et al., 2012; Lewis et al., 2015; Kim et al., 2020), although some controversy remains about the relative importance of PAD4 for ETs release (Neeli and Radic, 2013; Kenny et al., 2017; Claushuis et al., 2018; Guiducci et al., 2018; Silva et al., 2019; Thompson-Souza et al., 2020). Thus, using fluorimetry (Figure 7A) and confocal fluorescence microscopy (Figure 7B), we observed that in the presence of GSK484 (10 μM), a selective PAD4 inhibitor, EETs release in response to *A. fumigatus* was not inhibited (cell: fungal ratio of 1:10, 6 h). Citrullinated histone H3 expression was increased in the presence of *A. fumigatus* and negatively modulated in the presence of GSK484 (10 μM), as assessed by immunoblotting (Figure 7C). Interestingly, similar EET-like structures were observed in eosinophils pretreated with the PAD4 inhibitor and further stimulated with *A. fumigatus*; however, citrullinated histone H3 sites were not detectable (Figure 7D, EETs stained by Hoechst in blue and by anti-citrullinated histone H3 antibodies in green).

### Fungus Viability Is Dispensable for *A. fumigatus*-Induced EETs Release

**FIGURE 8:**
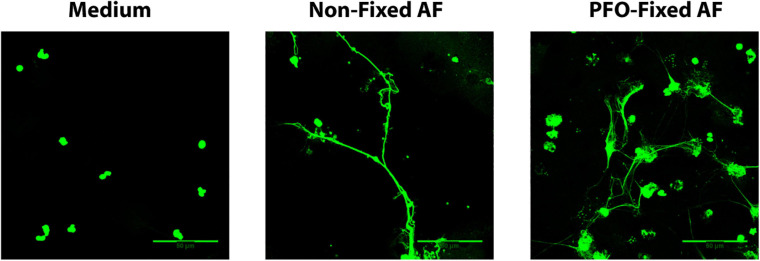
Fungus viability is dispensable for *A. fumigatus*-induced EETs release. Human eosinophils were stimulated with both viable and paraformaldehyde (PFO)-fixed *A. fumigatus* conidia (cell: fungus ratio of 1:10, 6 h). Samples were analyzed by confocal fluorescence microscopy after staining for DNA (Sytox Green, green). Representative images of 4 independent experiments (*n* = 4). AF = *Aspergillus fumigatus.*

To evaluate whether *A. fumigatus* conidia viability is crucial for the process of EETs release, we stimulated eosinophils with both viable and PFO-fixed *A. fumigatus* conidia. As shown in Figure 8, eosinophils responded by releasing EETs when co-cultured with either live or fixed *A. fumigatus* conidia (cell: fungal ratio of 1:10, 6 h).

## Discussion

**FIGURE 9:**
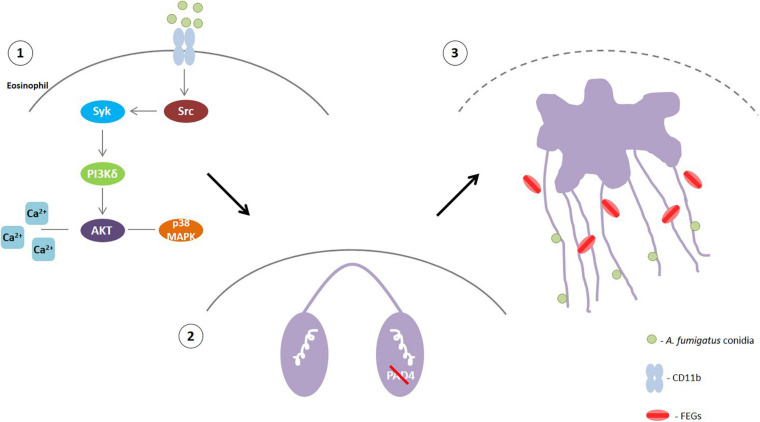
**(1)** *A. fumigatus* conidia-human eosinophil interaction triggers the activation of Src that will phosphorylate Syk tyrosine kinases. Then, PI3δK will be activated followed by Akt, probably with the participation of calcium. p38 MAPK will also likely be activated downstream the PI3K signaling **(2)** This signaling leads to histone citrullination mediated by PAD4 and chromatin decondensation. However, PAD4 and H3 citrullination are not essential for EETs extrusion. **(3)** Rupture of the nuclear envelope and plasma membrane allowed the release of chromatin-formed EETs that entrap *A. fumigatus* conidia and free extracellular granules (FEGs).

Eosinophils are the major leukocytes involved in ABPA, which is the most prevalent fungal allergic manifestation among ABPMs (Shah and Panjabi, 2002; Chowdhary et al., 2014). Thus, studies that contribute to a better understanding of ABPA pathology and can elucidate possible therapeutic targets for patient treatment are extremely relevant. Previous studies have shown that EETs are present in the mucus plugs of ABPA patients and that human eosinophils release EETs in response to *A. fumigatus* conidia *in vitro* (Muniz et al., 2018; Ueki et al., 2018). Nevertheless, efforts to understand eosinophil-*A. fumigatus* recognition and the mechanisms that drive this interaction are still in progress. Here, we demonstrated by both TEM and SEM that eosinophils responding to *A. fumigatus* stimulation present prominent morphological alterations typical of EETosis. Moreover, *A. fumigatus*-induced EETs release depends on the calcium, Src, PI3K, p38 MAPK and Akt signaling pathways. Interestingly, we determined that human eosinophils release EETs in response to *A. fumigatus* independently of PAD4 and histone citrullination through a process in which fungus viability is dispensable (Figure 9).

EETosis with a cytolytic profile and extrusion of extracellular traps has increasingly been identified in several eosinophilic diseases (Ueki et al., 2016a, b, 2018). This process of cell death associated to ETs release is morphologically distinct from other classic cell death processes, such as apoptosis and necrosis (Fuchs et al., 2007; Brinkmann and Zychlinsky, 2012), and has been associated with different leukocytes, especially neutrophils (Brinkmann et al., 2004), mast cells (von Kockritz-Blickwede et al., 2008) and eosinophils (Ueki et al., 2013). Here, we used high-resolution TEM to characterize intracellular events associated with EETosis. Our TEM findings revealed that *A. fumigatus*-stimulated eosinophils elicited nuclear changes typical of EETosis (decondensation, delobulation/rounding, disruption of nuclear envelope, chromatin expansion in the cytoplasm and further release of EETs) (Figure 1). These processes are well characterized in neutrophils (Fuchs et al., 2007), but are poorly understood in human eosinophils. In contrast to the process of ETosis in neutrophils (termed NETosis), which leads to the release of granule contents mixed with released chromatin (Brinkmann et al., 2004; Fuchs et al., 2007), EETosis enables the release of clusters of FEGs, with preserved morphology and contents, together with EETs. Our ultrastructural results showing FEGs by both TEM (Figures 1C, 2D) and SEM (Figures 2A,Ai) are in accordance with previous works demonstrating that EETosis induced by different stimuli, including the lipid mediator lysophosphatidylserine and *A. fumigatus* conidia, occurs in the presence of punctual MBP labeling (indicative of intact granules) and in the absence of linear immunostaining for MBP or ECP (indicative of disrupted granules) (Ueki et al., 2013; Muniz et al., 2018; Kim et al., 2020). This means that distinct mechanisms are operating in neutrophils and eosinophils in the process of granule product release in association with DNA traps during ETosis. For eosinophils, FEGs are secretory-competent organelles acting as “cluster bombs” that selectively release proteins under specific stimuli (Neves et al., 2008; Ueki et al., 2013). However, the consequences of these secretory entities associated with EETs still require clarification.

The capacity of chromatin to actively participate in NETosis is now recognized (Neubert et al., 2018), in addition to its classical role in regulating gene expression. During NETosis, after certain signaling events and enzymatic reactions (including histone citrullination and phosphorylation events), the chromatin expands, which drives the rupture of the nuclear envelope, determining the point of no return. Here, we noted the same event for human eosinophils undergoing EETosis. Although signaling molecules are usually the focus of studies on cell activation processes, recent works have demonstrated that chromatin changes are crucial in determining cell fate (Neubert et al., 2018). Thus, our ultrastructural observations bring a new appreciation of the importance of linking intracellular signaling pathways to nuclear entities and structural protein modifications to better delineate a stimulus-specific cell activation process.

We previously demonstrated that intracellular signaling involved in the release of EETs in response to *A. fumigatus* is Syk- and CD11b β2-integrin-dependent (Muniz et al., 2018). Src and Syk kinases have been implicated in the cell signaling of various classes of receptors involved in fungal molecule recognition, including integrins (Mocsai et al., 2002, 2006; Jakus et al., 2007) and C-type lectins (Rogers et al., 2005; Kerrigan and Brown, 2010). Src-family kinases are involved in β_2_ integrin-mediated responses in which the Src kinases are implicated in Syk phosphorylation in neutrophils (Mocsai et al., 2006). In line with these findings, our results reveal that *A. fumigatus*-induced EETs release is blocked in the presence of a Src inhibitor. Additionally, in agreement with our findings, neutrophils respond to *A. fumigatus* conidia by releasing NETs in a Syk-Src-dependent signaling pathway (Silva et al., 2019).

PI3Ks are enzymes that catalyze the phosphorylation of one or more inositol phospholipids in the 3-position of the inositol ring. PI3Ks can be divided into classes I, II, and III. Class IA PI3Ks, which include PI3K α, β, and δ, are activated by stimulation of tyrosine kinase-based receptors. PI3Kγ, the only member of class IB, is activated by stimulation of GPCR subunits (Vanhaesebroeck et al., 2010). We found that both a non-selective (wortmannin) and a class I-selective (AS605240, 10 μM) PI3K inhibitor blocked *A. fumigatus*-induced EET release. Because there have been many studies of wortmannin as a non-selective inhibitor of the different PI3K classes and of other possible targets (Makni-Maalej et al., 2013; Germic et al., 2017), we additionally used the compound AS605240. AS605240 at 10 μM has been reported as an inhibitor of class I PIKs but is unable at this concentration to distinguish between class A (PI3K α, β and δ) and B (PI3Kγ) (Sadhu et al., 2003; Silva et al., 2019). In mammals, class I PI3Ks are present in all cell types, including eosinophils, with PI3Kδ and γ highly enriched in leukocytes (Kok et al., 2009). As mentioned, we have previously described the dependence of *A. fumigatus*-induced EET release on Syk signaling (Muniz et al., 2018). Syk is a tyrosine kinase that is crucial in the signaling pathways mediated by Dectin-1 and the β_2_ integrin CD11b/CD18 (Mocsai et al., 2002; Rogers et al., 2005). Taking in account that receptors utilizing protein tyrosine kinase activation are known to be more related to the activation of class IA PI3Ks (PI3K α, β, and δ), we investigated the role of class I PI3K δ in *A. fumigatus*-induced EET release. We observed that compound IC-87114 at 1 μM (concentration known to be selective for the class I δ isoform) (Sadhu et al., 2003; Silva et al., 2019) completely inhibited EETs release. Indeed, the importance of PI3K δ in eosinophils and in experimental models of allergic inflammation has been previously reported (Lee et al., 2006; Nashed et al., 2007; Kang et al., 2012). Kang and colleagues demonstrated that treatment with ICI87114 reduced murine bone marrow-derived eosinophil adhesion and Mac-1 expression and had inhibitory effects on eotaxin-1-induced chemotaxis and shape change (Kang et al., 2012). However, the authors used 10 μM ICI87114. In our studies, we used 1 μM ICI87114. Some studies suggest that 10 μM ICI87114 might also have prominent effects on PI3Kγ (Sadhu et al., 2003; Silva et al., 2019). Regarding the PI3Kγ isoform, some important effects have also been described in human eosinophils but are related to the activation of GPCRs (Saito et al., 2014). Saito and colleagues described the effects of specific inhibition of the PI3Kγ isoform on human eosinophil chemotaxis, adherence and degranulation induced by eotaxin (Saito et al., 2014). Since PI3Kγ signaling is associated with GPCRs (such as CCR3), whereas PI3K α, β, and δ are activated by receptor tyrosine kinase, it is possible that PI3Kγ and δ isoforms might have distinct roles in eosinophil intracellular signaling and function depending on the stimuli and the cognate receptor involved. In fact, PI3K activation has been implicated as crucial for ROS generation-induced DNA trap release in eosinophils and neutrophils stimulated with GM−CSF and C5a or with low concentrations of PMA (Germic et al., 2017); and in neutrophils stimulated with immobilized immune complexes (Behnen et al., 2014). In contrast, other studies have pointed that ROS and PI3K is dispensable for lysophosphatidylserine-induced EETs extrusion (Kim et al., 2020). The PI3K δ isoform specifically has been described as critically involved in NETs release in response to *Leishmania amazonensis* (DeSouza-Vieira et al., 2016) and *A. fumigatus* (Silva et al., 2019) in a signaling pathway upstream of ROS production. In contrast, the PI3Kγ isoform is not involved in *A. fumigatus*-induced NETs release (Silva et al., 2019).

The activation of Akt has been reported to be the downstream target of PI3K in various cells, including eosinophils (Alessi et al., 1997; Machida et al., 2005; Hawkins et al., 2010). The participation of Akt has been demonstrated in the context of NETs release following immunocomplex recognition and signaling via Mac-1 (Behnen et al., 2014). Moreover, it has been demonstrated that PMA-induced NETs formation is dependent on Akt activation, which suppresses apoptosis via the inhibition of caspases (Douda et al., 2014). In agreement with these findings, we found that the release of EETs in response to *A. fumigatus* requires the PI3 kinase and Akt signaling pathways. Behnen and colleagues showed that CR3 (CD11b/CD18) activation through the recognition of immunocomplexes by the FcγRIIIB receptor induces NETs release via the Src/Syk, PI3K/Akt, p38 MAPK, and ERK1/2 signaling pathways (Behnen et al., 2014). The involvement of p38 MAPK in NETs release induced by different stimuli, such as bacteria or PMA, was previously reported (Keshari et al., 2013; Behnen et al., 2014; Douda et al., 2015; Ma et al., 2018). In agreement with these studies, we found that the release of EETs was inhibited when eosinophils were pretreated with SB202190, suggesting the involvement of p38-MAPK signaling in *A. fumigatus*-induced EETs release.

As we observed that *A. fumigatus*-induced EETs release triggered the Src/Syk, PI3K/Akt and p38 MAPK signaling pathways, we also examined calcium. Changes in leukocyte calcium levels have been consistently related to several leukocyte functions, including cell adhesion, chemotaxis and degranulation, among others (Dixit and Simon, 2012; Hann et al., 2020). The relationship between calcium and ETosis has been supported by different studies in eosinophils, but mostly by studies in neutrophils (Parker et al., 2012; Gupta et al., 2014; Ueki et al., 2016a; Kenny et al., 2017; de Bont et al., 2018). Moreover, the activation of Akt has been found to be regulated by an elevation of calcium during the NETosis process (Douda et al., 2014). Thus, our finding that the calcium chelator BAPTA-AM inhibited EETs release induced by *A. fumigatus* corroborates these previous observations. Ueki and colleagues found that EDTA inhibited the EETs extrusion induced by A23187, PMA, or immobilized IgG in human eosinophils (Ueki et al., 2016a). In neutrophils, Kenny and colleagues demonstrated the relevance of intracellular calcium for PMA-induced NET production by showing a strong inhibition of NETosis after treating neutrophils with BAPTA-AM (Kenny et al., 2017). In agreement with this observation, another work also provided evidence that external calcium is dispensable for NETs extrusion induced by PMA (Douda et al., 2015). In contrast, a different study showed that IL-8-mediated NET formation requires calcium fluxes from both intracellular and extracellular pools, while only extracellular calcium appeared to be important for PMA-mediated NET generation (Gupta et al., 2014). Accordingly, ionomycin and other calcium ionophores have been widely used as known inducers of NETs and EETs (Douda et al., 2015; Ueki et al., 2016a). In this context, it is clear that calcium plays a crucial role in *A. fumigatus*-induced ETTs extrusion. However, whether calcium is important for Akt activation during this EETosis process or which is the major source of calcium (intracellular or extracellular or both) are questions still to be answered.

Although several studies have implicated a role for PAD4 in histone citrullination and chromatin decondensation in the process of ETosis (Wang et al., 2009; Li et al., 2010; Lewis et al., 2015; Van Avondt and Hartl, 2018; Kim et al., 2020), others have suggested that PAD4 might not be essential for this process even when H3 citrullination is reduced (Neeli and Radic, 2013; Kenny et al., 2017; Claushuis et al., 2018; Guiducci et al., 2018; Silva et al., 2019; Thompson-Souza et al., 2020). According to most ET studies, the citrullination of histone 3 by the enzyme PAD4, expressed in the nuclei of eosinophils and other granulocytes (Asaga et al., 2001; Nakashima et al., 2002; Kim et al., 2020), results in weakened DNA–histone binding, thereby facilitating the release of DNA from the nucleus and out of the cell (Van Avondt and Hartl, 2018). However, other studies have shown that histone citrullination-independent mechanisms occur in the process of NETs release in response to *Candida albicans* (Guiducci et al., 2018), bacteria (*Streptococcus* and *Klebsiella pneumoniae*) (Kenny et al., 2017; Claushuis et al., 2018), *Histoplasma capsulatum* (Thompson-Souza et al., 2020) and *A. fumigatus* (Silva et al., 2019). Under our conditions, we observed that EETs generated in response to *A. fumigatus* exhibited histone citrullination that was PAD4-dependent; however, PAD4 and H3 citrullination are not essential for EETs extrusion. In fact, the existence of NET release pathways independent of histone citrullination has been described for PMA, one of the most recognized promoters of NET release (Neeli and Radic, 2013; Kenny et al., 2017). In this context, the dependency of ETs extrusion on PAD4 and histone citrullination is questionable and might depend on the stimuli and cell type involved. One question that remains in eosinophils is which molecule is responsible for chromatin decondensation, since it can occur independently of PAD4 and histone citrullination. In neutrophils, elastase and myeloperoxidase have been described as important for this process (Papayannopoulos et al., 2010), but in eosinophils, this is a point for future investigations. Moreover, the consequences of the release of non-citrullinated EETs for the host immune response, for the fungus, and for ABPA development remain unknown. We previously showed that EETs do not contribute to the killing and impairment of *A. fumigatus* conidia (Muniz et al., 2018). Thus, the impact of non-citrullinated EETs on fungal viability is potentially not critical. However, other consequences of these non-citrullinated EETs regarding ABPA development and the host immune response cannot be discarded. In a study of *Klebsiella pneumoniae*–induced pneumonia, NETs formed in the absence of PAD4 and histone citrullination did not affect bacterial growth or lung inflammation (Claushuis et al., 2018). Whether the same is valid for eosinophils or eosinophilic lung inflammation in the context of ABPA remains to be addressed. Our findings also demonstrated that *A. fumigatus* is capable of inducing EETs release independent of fungal viability. In previous works, dead *A. fumigatus* conidia were also capable of inducing neutrophils to release extracellular DNA traps (Bruns et al., 2010; McCormick et al., 2010). Thus, fungal cell metabolism seems to play no active role in this interaction, both in EETs and NETs formation.

In this study, we identified several components that drive EETs release in response to the fungus *A. fumigatus*. Based on this and previous studies (Muniz et al., 2018), we believe that the excessive release of EETs may contribute to the formation of sticky mucus, which in turn contributes to airway obstruction and lung function impairment. Furthermore, because EETs lack killing activity against *A. fumigatus* (Muniz et al., 2018), their association with clusters of functional FEGs may potentiate the pro-inflammatory roles of these structures. Thus, therapeutic interventions to avoid or degrade these DNA traps may represent an interesting approach to minimize inflammatory lung damage without major impacts on fungal clearance. In addition to clarifying mechanisms that underlie the interaction between *A. fumigatus* and eosinophils, these findings may help improve our understanding of ABPA pathogenesis and treatment.

## Data Availability Statement

The original contributions presented in the study are included in the article/supplementary material, further inquiries can be directed to the corresponding author/s.

## Ethics Statement

The studies involving human participants were reviewed and approved by Committee on Human Research at Clementino Fraga Filho Hospital (Federal University of Rio de Janeiro). The patients/participants provided their written informed consent to participate in this study.

## Author Contributions

MVB, IG, MAAD, GAT-S, VSM, and CRIV conducted the experiments and acquired and analyzed the data. MVB, RCNM, RTF, and JSN designed the research studies, analyzed the data and provided reagents. MVB and JSN wrote the manuscript. RCNM and RTF critically revised the manuscript for important intellectual content. All authors contributed to the article and approved the submitted version.

## Conflict of Interest

The authors declare that the research was conducted in the absence of any commercial or financial relationships that could be construed as a potential conflict of interest.
